# ^13^C metabolic flux analysis: Classification and characterization from the perspective of mathematical modeling and application in physiological research of neural cell

**DOI:** 10.3389/fnmol.2022.883466

**Published:** 2022-09-08

**Authors:** Birui Tian, Meifeng Chen, Lunxian Liu, Bin Rui, Zhouhui Deng, Zhengdong Zhang, Tie Shen

**Affiliations:** ^1^Key Laboratory of Information and Computing Science Guizhou Province, Guizhou Normal University, Guiyang, China; ^2^Key Laboratory of National Forestry and Grassland Administration on Biodiversity Conservation in Karst Mountainous Areas of Southwestern China, Key Laboratory of Plant Physiology and Development Regulation, School of Life Science, Guizhou Normal University, Guiyang, China; ^3^Eurofins Lancaster Laboratories Professional Scientific Services, Lancaster, PA, United States; ^4^China Guizhou Science Data Center Gui’an Supercomputing Center, Guiyang, China; ^5^College of Mathematics and Information Science, Guiyang University, Guiyang, China

**Keywords:** metabolic flux analysis, isotope labeling model, ^13^C fluxomics, isotope tracing, neural cell

## Abstract

^13^C metabolic flux analysis (^13^C-MFA) has emerged as a forceful tool for quantifying *in vivo* metabolic pathway activity of different biological systems. This technology plays an important role in understanding intracellular metabolism and revealing patho-physiology mechanism. Recently, it has evolved into a method family with great diversity in experiments, analytics, and mathematics. In this review, we classify and characterize the various branch of ^13^C-MFA from a unified perspective of mathematical modeling. By linking different parts in the model to each step of its workflow, the specific technologies of ^13^C-MFA are put into discussion, including the isotope labeling model (ILM), isotope pattern measuring technique, optimization algorithm and statistical method. Its application in physiological research in neural cell has also been reviewed.

## Introduction

Metabolic flux refers to the *in vivo* conversion rate of metabolites, including the rate of the enzymatic reaction and the transport rate between different compartments (Hui et al., 2020). Flux information deepens our understanding of cell growth and maintenance in response to environmental changes (Becker and Wittmann, 2018; O’sullivan et al., 2019; Van Gastel and Carmeliet, 2021). It is also crucial for revealing the sites and mechanisms of metabolic regulation (Zamboni et al., 2015; Mei et al., 2021).

Accurately estimating flux within complex metabolic networks requires ^13^C metabolic fluxomics (Niedenführ et al., 2015; Varanasi et al., 2019; Lawson et al., 2021). ^13^C metabolic fluxomics has been applied to a number of important studies in recent years, strongly pushing forward the frontiers of metabolic research (Varanasi et al., 2019; Van Gastel and Carmeliet, 2021). This technique can identify changes in metabolic pathway activity and discover novel metabolic pathways (Zhang et al., 2018; Wang et al., 2020; Cobbold et al., 2021). Therefore, it is widely used to reveal metabolic changes in various pathogenic processes, such as colorectal adenocarcinomas (Wang et al., 2018), diabetes (Neinast et al., 2019), retinal degenerative disease (Yam et al., 2019) and immune cells (Varanasi et al., 2019). The technique can characterize the metabolic features of multiple plant organs, such as maize embryos (Cocuron et al., 2019), *Arabidopsis* leaves (Ma et al., 2014) and developing *camelina* seeds (Carey et al., 2020). It has also been applied in metabolic engineering to guide the optimization of the synthesis of target products, such as acetaldehyde (Cheah et al., 2020), isopropanol (Okahashi et al., 2017) and vitamin B2 (Schwechheimer et al., 2018).

In this review, metabolic fluxomics methods were first classified from the perspective of data modeling. Then, we introduced the components of fluxomics, including the modeling framework, experimental measuring technique, and optimization techniques. Finally, the application of ^13^C metabolic flux analysis (^13^C-MFA) in neural cell was reviewed.

## Classification of ^13^C metabolic fluxomics

**FIGURE 1:**
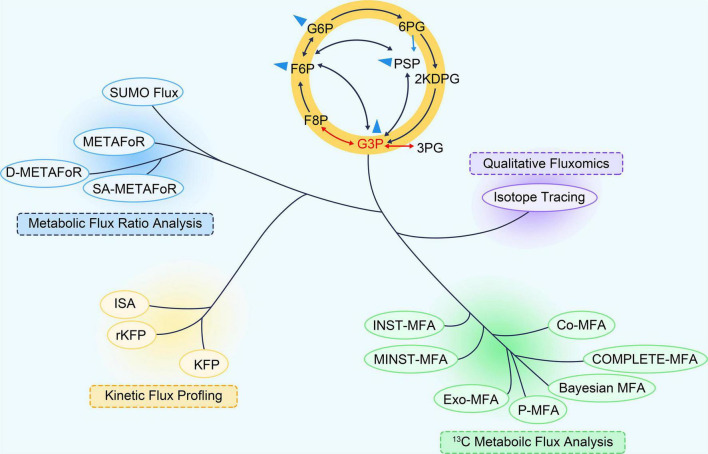
The “phylogeny” of ^13^C fluxomics methods. A phylogenetic tree can describe the relationship and application of these methods. Co-MFA, Co-culture Metabolic Flux Analysis; COMPLETE-MFA, Complementary parallel labeling experiments technique for Metabolic Flux Analysis; D-METAFoR, Dynamic Metabolic Flux Ratio Analysis; Exo-MFA, by Exosome-mediated Metabolic Flux Analysis; INST-MFA, Isotopically Non-stationary ^13^C Metabolic Flux Analysis; ISA, Isotopic Spectral Analysis; KFP, Kinetic Flux Profiling; METAFoR, Metabolic Flux Ratio Analysis; MNST-MFA, Metabolically non-stationary ^13^C metabolic flux analysis; P-MFA, Parsimonious Metabolic Flux Analysis; rKFP, relative Kinetic Flux Profiling; ScalaFlux, Scalable Metabolic Flux Analysis.

Recently, ^13^C-based metabolic fluxomics have evolved into a large family of diverse methods as shown in Figure 1 and Table 1 (Niedenführ et al., 2015). The major categories are as follows:

**TABLE 1:** Comparison of different fluxomics method.

Method type		Applicable scene	Computational complexity	Limitation
Qualitative fluxomics (isotope tracing)		Any system	Easy	Just local and qualitative value
Metabolic flux ratios analysis		Systems where flux, metabolites, and their labeling are constant	Medium	Just local and relative quantitative value
Kinetic flux profiling		Systems where flux, metabolites are constant while the labeling is variable	Medium	Just local and relative quantitative value
Metabolic flux analysis	Stationary state13C metabolic flux analysis	Systems where flux, metabolites and their labeling are constant	Medium	Not applicable to dynamic system
	Isotopically instationary 13C metabolic flux analysis	Systems where flux, metabolites are constant while the labeling is variable	High	Not applicable to metabolically dynamic system
	Metabolically instationary 13C metabolic flux analysis	Systems where flux, metabolites and labeling are variable	Very high	Hard to perform

### Qualitative fluxomics (isotope tracing)

In qualitative fluxomics, an isotope-labeled tracer is incorporated into the metabolic system, leading to variation in the isotopic pattern of the metabolites (Faubert et al., 2017; Jang et al., 2018). Qualitative pathway activity changes can be deduced by comparing isotopic data (Ma et al., 2017; Liang et al., 2021). For instance, feeding labeled glucose results in M+3 triose phosphates. M+3 fructose bisphosphate reflects the reversibility of aldolase, while M+3 glucose-6-phosphate reflects fructose bisphosphatase activity (Hackett et al., 2016).

### ^13^C flux ratios

Based on the differences between the isotopic compositions of the metabolic precursor and the product, the relative fraction of metabolic fluxes converging to a node can be directly calculated (Sauer et al., 1999; Nanchen et al., 2007). A dozen such ratios can be identified from the isotope labeling patterns of amino acids or organic acids (Shen et al., 2013). This ratio estimation method can be performed when isotope labeling is dynamic (Hörl et al., 2013). Currently, the ratios can be estimated from ^13^C measurements by dedicated machine learning predictors (Kogadeeva and Zamboni, 2016). The metabolic flux ratio (FR) method has a unique advantage when the overall network topology is unclear and metabolite outflow rate measurements are difficult to detect and determine (Rantanen et al., 2008).

### ^13^C kinetic flux profiling

The kinetic flux profiling (KFP) method assumes that the labeled fraction of the metabolite pool changes exponentially during the labeling process. As long as the pool size is accurately measured, this method can estimate the absolute flux through sequential linear reactions according to the kinetic elution equation (Yuan et al., 2008). KFP is extended for quantifying fluxes within subnetworks encompassing convergent nodes and bounded by a unidirectional linear reaction (Szecowka et al., 2013; Heise et al., 2014). It was used to detect kinetic parameters such as the incorporation rate of [6-^13^C] glucose into phospholipids and the turnover rate of acylglycerol to determine the effect of deleting the cg6718 gene in *Drosophila melanogaster* (Schlame et al., 2020).

### ^13^C metabolic flux analysis

As a major component of metabolic fluxomics, ^13^C-MFA can accurately determine the absolute value of the flux of the global metabolic network (Antoniewicz, 2015), making it a unique tool for metabolic research. In the carbon labeling experiment, the isotopic distribution of these metabolites depends on the isotopic distribution of the substrate and metabolic flux values (Cheah and Young, 2018). Flux values can be estimated after the isotopic labeling values of measured metabolites are optimally fitted. The flux estimation process can be formalized as the following optimization problem:


(1) $$\begin{array}{c} arg\;min:\left(x-x^{M}\right)\Sigma_{\varepsilon }{\left(x-x^{M}\right)}^{T}\\ \;s.t.S\cdot v=0\\ \;M\cdot v\geq b\\ \;A_{1}\left(v\right)X_{1}-B_{1}Y_{1}\left(y_{1}^{in}\right)=\frac{dX_{1}}{dt}\\ \;A_{2}\left(v\right)X_{2}-B_{2}Y_{2}\left(y_{2}^{in},X_{1}\right)=\frac{dX_{2}}{dt}\\ \;\vdots \\ \;A_{n}\left(v\right)X_{n}-B_{n}Y_{n}\left(y_{n}^{in},X_{n-1,\ldots ,}X_{1}\right)=\frac{dX_{n}}{dt} \end{array}$$

*v* represents the vector of the metabolic flux, *s* represents the stoichiometric matrix of the metabolic network, and M⋅*v* ≥ *b* provides additional constraints from physiological parameters or excretion metabolite measurement. *y_*i*_*^in^** represents vectors of the isotope labeled substrate. *X*_*n*_ is a matrix, and its rows are the vectors of the isotope labeling model (ILM) of the corresponding elementary metabolite unit (EMU) fragment with *n* carbon atoms. *Y*_*n*_ is a matrix similar to *X*_*n*_, in which its rows are the vectors of the ILM of the corresponding input substrate and/or the calculated EMU fragment with *1 ∼ (n-1)* carbon atoms. In the objective function, *x* is the vector of the isotope-labeled molecules in *X*_1_,…, *X*_*n*_, and *x^M^* is the experimental counterpart to *x*. *A*_*n*_ and *B*_*n*_ represent the system matrix determined by the corresponding metabolic reaction topology and atomic transfer relationship. Σ_*e*_ represents the covariance matrix of the measured values.

**FIGURE 2:**
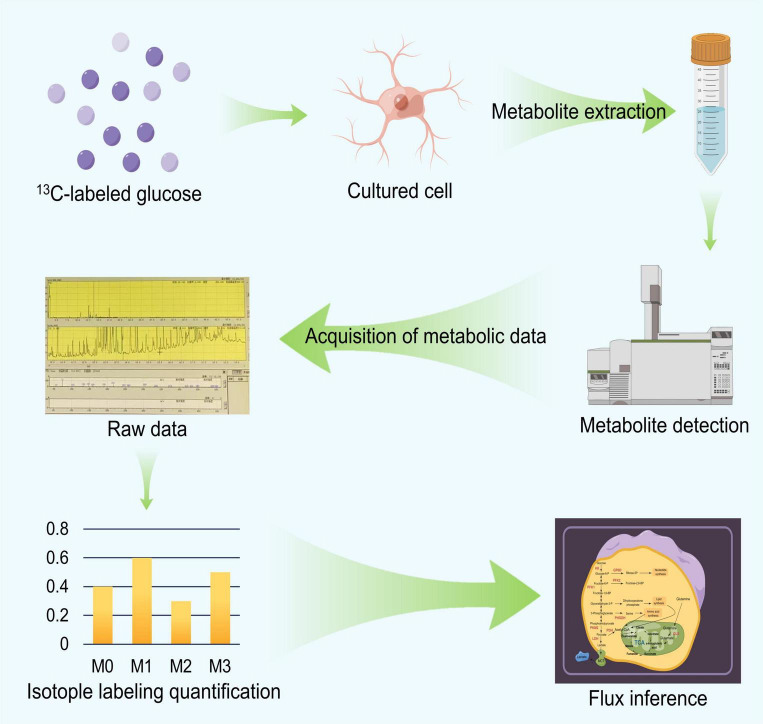
The flow chart of ^13^C Metabolic Flux Analysis (^13^C MFA).

We briefly described the workflow and principles of ^13^C-MFA as depicted in Figure 2 and then discussed the technical details used in the process. The ^13^C-MFA method first involves a carbon labeling experiment (Hollinshead et al., 2019). Specific ^13^C-labeled substances are chosen as carbon sources for cell culture experiments, depending on the cell type. For example, early ^13^C-MFA approaches often used various mixtures of [1-^13^C] glucose, [U-^13^C] glucose and unlabeled glucose as substrates (Leighty and Antoniewicz, 2013). In carbon labeling experiments, the isotope label material is gradually distributed to various metabolites in the metabolic pathway. Since the amount and location of ^13^C in metabolites are closely related to metabolic flux, different metabolic flux distributions produce different isotope labeling levels. Then, the labeling status of the substrate is determined based on a specific mathematical relationship between the metabolic flux distribution and the isotopic labeling status of the metabolites *in vivo* (Wang et al., 2020), which can be described by formula (1). Based on this relationship, we can obtain the distribution of the metabolic flux by accurately measuring the isotope labeling levels of the metabolites. The accurate method used for determining the status of the ^13^C isotope label includes mass spectrometry (GC−MS and LC−MS) and nuclear magnetic resonance (NMR) spectroscopy (Rahim et al., 2022). Then, we can estimate the level of metabolic flux. Specifically, we can first determine the random distribution of the metabolic flux, and then, based on the value from formula (1), we can calculate the corresponding theoretical isotope labeling status of each metabolite. Then, the calculated labeling status is compared with the measured labeling status. According to the difference between the two, the given metabolic flux distribution can be repeatedly adjusted until the difference between the two is less than a specific threshold. The resulting metabolic flux distribution is the true distribution of the estimates (Weitzel et al., 2013).

### Classification of ^13^C metabolic flux analysis

**FIGURE 3:**
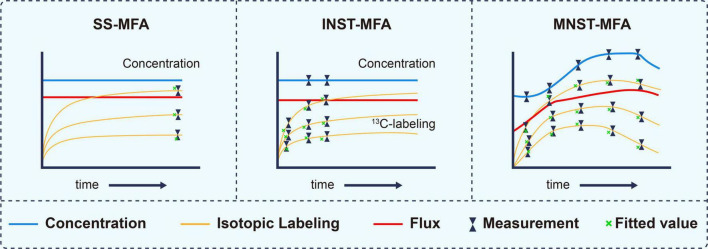
Characterization of different ^13^C metabolic flux analysis methods, modified from (37). The essential distinguishing feature between these methods are whether isotopically stationary state is reached and whether metabolic steady state is assumed. SS-MFA, Stationary State ^13^C Metabolic Flux Analysis; INST-MFA, Isotopically Non-stationary ^13^C Metabolic Flux Analysis; MNST-MFA, Metabolically non-stationary ^13^C metabolic flux analysis.

According to formula (1), metabolic flux analysis can be divided into three categories, stationary state^13^C metabolic flux analysis (SS-MFA) (Weitzel et al., 2013), isotopically instationary ^13^C metabolic flux analysis (INST-MFA) (Wahl et al., 2008; Young et al., 2008) and metabolically instationary ^13^C metabolic flux analysis (MNST-MFA) in Figure 3 (Abate et al., 2012; Van Heerden et al., 2014).

SS-MFA refers to the case when all d*X*_*i*_/dt of the constraint conditions in formula (1) are equal to 0 and *v* is constant. The method is suitable for systems in which metabolic flux and isotope labeling state do not change with time (Wiechert and Nöh, 2005). Such system is usually at end of a long-term labeling process and is also called stable labeling. The flux distribution can be deduced by measuring the isotopic labeling state of metabolites in the steady state.

INST-MFA refers to the case when not all d*X*_*i*_/dt in the constraint conditions in formula (1) are equal to 0 and *v* is constant. It can be applied to systems in which the metabolic flux and metabolite concentration do not change with time while the isotopic labeling fraction changes with time (Gopalakrishnan et al., 2018). In such a system, the isotope labeling process is in an early dynamic stage and does not reach a stationary state. Therefore, the labeling state is a function of not only the substrate labeling fraction and metabolic flux but also the labeling time. It is necessary to carry out multiple dynamic measurements and to solve the isotopic differential equations to deduce the metabolic flux.

MNST-MFA refers to the case when not all d*X*_*i*_/dt in the constraint conditions in formula (1) are equal to 0 and *v* is a time-varying function. This method fits well with systems in which the metabolite concentration, metabolic flux and isotope labeling fraction all change with time (Abate et al., 2012). Its output is not just a flux value but a flux profile over a period of time (Quek et al., 2020).

## Technology of ^13^C metabolic flux analysis

The necessary technology of ^13^C-MFA includes isotope labeling modeling, isotope labeling state measurement, flux optimization and statistical analysis. The details are as follows:

### Isotope labeling model

The quantitative interpretation of isotope-labeled data, that is, the estimation of metabolic flux, requires an algorithm that can describe the accurate relationship between metabolic flux and isotope-labeled fractions (Zamboni, 2011). We call this the ILM, that is, the content of *X* in formula (1). The same ^13^C labeling experiment can be simulated with different ILMs (Weitzel et al., 2007). Different ILMs characterize the same labeling system from different perspectives.

**FIGURE 4:**
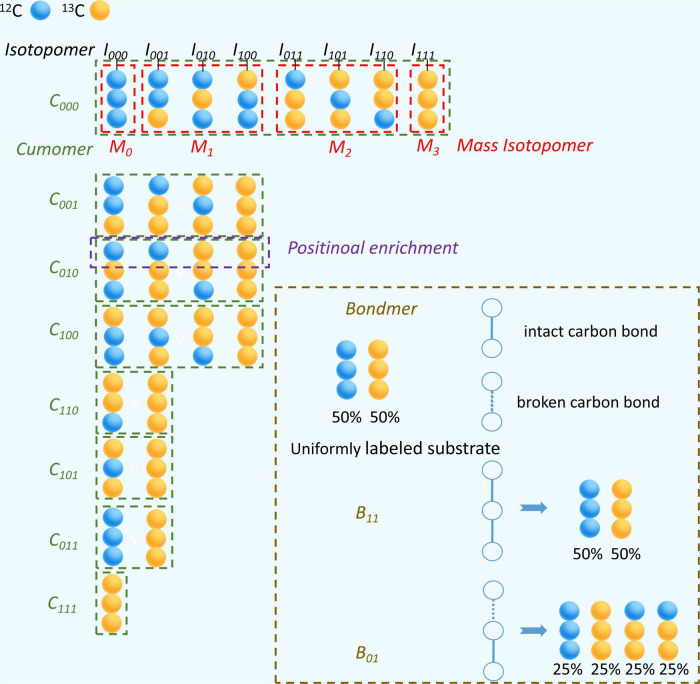
Characterization of different ^13^C isotope labeling model with a 3 carbon atoms metabolite. Blue ball represents ^12^C and yellow ball represents ^13^C. The isotope molecule associated with black line segment is specific type of isotopomer, such as I_100_. The isotope molecules in red dashed box belong to the same group of mass isotopomer, such as M_1_. The isotope molecules in green dashed box belong to the same group of cumomer, such as C_100_. In brown dashed box, white open circle represents a carbon position, blue represents a carbon-carbon bond that has never been broken by any reaction and blue dashed line represents a carbon-carbon bond that has been broken by a reaction and rejoined by another reaction. The percentage below a isotopic molecule is its abundance fraction.

If there is any ^13^C incorporated into the molecule, the same compound will be distinguished due to the number and position of ^13^C.^ 13^C isotopomer is any isomer of an organic compound differing only in the number and position of ^13^C (Table 2). Isotopomers corresponds to basic isotopically labeled molecules (Schmidt et al., 1997). The isotopomer I_100_ in Figure 3 represent a labeled molecule where the first carbon is ^13^C and the second and third carbons are ^12^C. Cumomers is another form of Isotopomer, and its coded appearance is exactly the same as that of Isotopomer. But the 0 in Isotopomer code means ^12^C, and the 0 in Cummer means “^12^C or ^13^C,” which is a collection where both ^12^C and ^13^C are allowed. Cumomers contain the same information as isotopomers, and the number of model particles is the same as that of isotopomers (Wiechert and De Graaf, 1997). The complete set of cumomers and the complete set of isotopomers are equivalent. The information of mass isotopomers and isotopic positional enrichment is coarser than that of isotopomers, and correspondingly, the number of model particles is lower than that of isotopomers (Hellerstein and Neese, 1992; Zupke and Stephanopoulos, 1994). Bondmer is an ILM in the case of uniformly labeled substrates. Each individual bondmer is a set of specific isotopomer whose fraction can calculated by a binomial distribution as shown in Figure 3 (Van Winden et al., 2002). The EMU framework is a framework suitable for simplifying and accelerating the simulation of various ILMs (Antoniewicz et al., 2007). It can accelerate various ILMs and has been widely adopted. Figure 4 shows the concept and corresponding relationship of various ILMs by taking a compound containing 3 carbon atoms as an example.

**TABLE 2:** Comparison of different isotope model.

Isotope model	Item number of *n* carbon compound	Feature
Cumomer	2*^n^*	Integrated in some software
Isotopomer	2*^n^*	Intuitive and direct description of isotope labeling
Bondmer	2^*n*–1^	Only applicable to uniformly labeled substrate

The cascade equation solution can reduce the computational complexity and has been used until now (Wiechert et al., 1997). Isotopic differential equations were developed as a basic framework for isotopically instationary flux analysis (Wahl et al., 2008; Young et al., 2008), which significantly expanded the scope of flux analysis. The modeling of mass isotopomers can be rearranged like that of cumomers and gradually parallelized (Zhang et al., 2020). In addition, a framework for metabolically instationary flux analysis was proposed and applied to the temporal flux reconfiguration of the glucose pathway of adipocytes in response to insulin (Abate et al., 2012; Quek et al., 2020).

### Isotope molecule measurement

The measurement of *X* in formula (1) determines the objective function. The appearance of ^13^C at different positions classifies a molecule as different isotopic molecules. Differences in the physical and chemical properties of ^13^C and ^12^C are the basis for discriminating these isotopic molecules. They have at least two differences. One is that ^13^C has a half integer nuclear spin, so it can be detected by NMR (Reardon et al., 2016). The other is that ^13^C has a greater mass number than ^12^C, so their difference can be detected by mass spectrometry.

**FIGURE 5:**
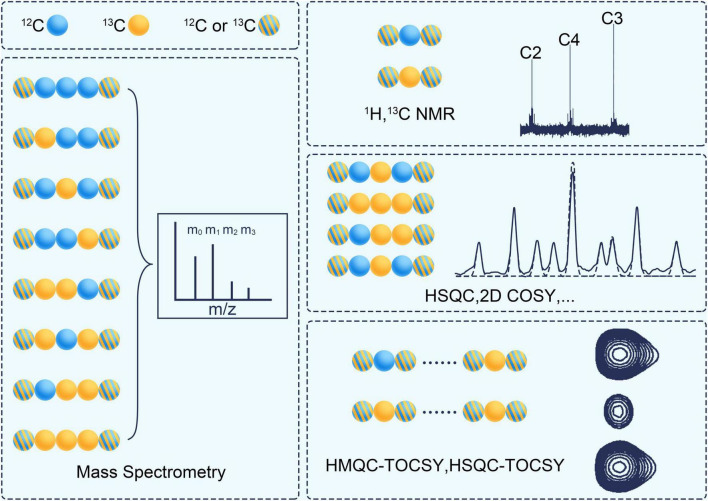
The analytical techniques for measuring the isotope labeling pattern. Blue ball represents ^12^C and yellow ball represents ^13^C. The stripped ball represents ^12^C or ^13^C. COSY, correlation spectroscopy; HSQC, heteronuclear single quantum coherence; HMQC, heteronuclear multiple quantum coherence; TCOSY, total correlation spectroscopy.

1D NMR of ^1^H and ^13^C can provide information on positional labeling enrichment as stated in ILM (Vinaixa et al., 2017; Deja et al., 2020; see Figure 5). Correlation Spectroscopy (COSY) is a 2D NMR technique that displays correlations between J-coupled nuclear by stepping up the delay between two 90°-proton pulses. Two-dimensional heteronuclear correlation spectroscopy (2D-COSY) (Szyperski, 1995), three-dimensional heteronuclear correlation spectroscopy (3D-COSY) (Boisseau et al., 2013) and proton-detected 2D heteronuclear single-quantum coherence (HSQC) (Lane et al., 2019) can detect the fraction of a specific subset of isotopomers. The set is a fragment of 3 continuous carbon atoms. Its middle carbon is ^13^C, while the carbons on both sides are ^12^C or ^13^C. 2D heteronuclear multiple quantum coherence-total correlation spectroscopy (HMQC-TOCSY) (Carvalho et al., 1998) and 3D total correlation-heteronuclear single-quantum coherence (TOCSY-HSQC) can detect another type subset of isotopomers (Reardon et al., 2016). This set is a fragment of continuous carbon atoms in the same spin system. One of its ends is ^13^C, and the other may be ^12^C or ^13^C. A non-uniform sampling technique can be used to improve the sensitivity and resolution of the fine structure in NMR spectrometry (Lee et al., 2017).

Gas chromatography-electron ionization (GC-EI)-quadrupole can quantify the mass isotopomers of amino acids (Dauner and Sauer, 2000), organic acids and phosphate sugars (Jung and Oh, 2015; Evers et al., 2021). With the development of tandem mass spectrometry, gas chromatography-electron ionization-triple quadrupole (GC-EI-tripleQ) (Okahashi et al., 2016), gas chromatography-chemical ionization- quadrupole time of flight (GC-CI-QToF) (Mairinger et al., 2015), liquid chromatography-electron spray ionization-triple quadrupole (LC-ESI-tripleQ) (Jeffrey et al., 2002; Rühl et al., 2012), and liquid chromatography-electron spray ionization-quadrupole time of flight (LC-ESI-QToF) (Kappelmann et al., 2017) have been used to measure position enrichment and mass isotopomers. Hundreds of metabolites can be detected, and more accurate results can be obtained. To simplify the sample processing, capillary electrophoresis QToF has also been added to the lineup (Toya et al., 2007). Gas chromatography-combination isotope ratio mass spectrometry (GC-C-IRMS) is suitable for the accurate determination of very low isotopic enrichment abundance (Yuan et al., 2010; Croyal et al., 2016). The data of mass spectrometry should be corrected for naturally occurring heavy isotopes before used by flux analysis (Jeong et al., 2021).

### Optimization technique

Flux estimation solves the entire optimization problem of formula (1). This process depends on local or global optimization methods. As a heuristic method, an evolutionary algorithm was employed early in the optimization of S-MFA (Wiechert, 2001). Some of its derivatives, such as the convex evolutionary algorithm and adaptive evolutionary algorithm, were introduced later (Chen et al., 2007; Yang et al., 2007). Simulated annealing has also been successfully applied to S-MFA (Fu et al., 2015).

A hybrid algorithm combining the trust-region method and sequential quadratic programming (SQP) facilitates numerically stable and accurate flux estimation of S-MFA (Yang et al., 2008). The Levenberg Marquardt algorithm, one type of trust-region method, is widely used and can guarantee fast convergence for INST-MFA (Young, 2014).

The sum-of-squares residual error (SSE) between the measured and simulated data is the main form of the objective function in formula (1)(Wiechert, 2001). Additionally, the Akaike information criterion (AIC) can be used as an objective function in seeking the simplest candidate models sufficient to generate the observed data (Alger et al., 2021).

### Flux uncertainty analysis

Since metabolic flux analysis is a parameter estimation problem, there are uncertainties in the obtained parameters. Flux uncertainty analysis should become part of routine analyses. Local linear error propagation based on the chain rule is an earlier method for determining the confidence interval of flux values (Wiechert et al., 1997). In contrast, a profile likelihood method would directly execute non-linear mapping over error propagation, which is preferred due to the corresponding high computational efficiency and accuracy (Antoniewicz et al., 2006). In addition to the error propagation method, another idea is to obtain the probability distribution of metabolic flux. The Monte Carlo method can be used to generate such a distribution based upon a Gaussian distribution or chi-square distribution assumption for the measurement error (Wittmann and Heinzle, 2002). In some experiments, the shape of this distribution can be posteriorly calculated using Markov chain Monte Carlo (MCMC) from isotopic data (Kadirkamanathan et al., 2006; Theorell et al., 2017).

### Derivative method

Different constraints can be introduced in formula (1) to generate a new derivative of MFA, which can expand the scope of metabolic flux analysis or make it more accurate in some cases. Compartment-specific metabolic flux in mitochondria and cytosol can be quantified by a spatial-flux analysis with rapid subcellular fractionation and quenching of metabolism (Lee et al., 2019). A flux analysis method for local subnetworks was recently proposed and needs only the information of the subnetwork of interest, requiring no additional knowledge of the surrounding networks (Millard et al., 2020). ^13^C MFA has also been extended to solve metabolic networks at the genome scale by incorporating a large set of secondary metabolic reactions (Blank et al., 2005; Martín et al., 2015; Gopalakrishnan et al., 2018). In addition, flux analysis goes beyond the scope of cells (Hui et al., 2020; Liu et al., 2020). The flux from source cells to recipient cells through vesicles can be accessed by exosome-mediated metabolic flux analysis (Exo-MFA) (Achreja et al., 2017). Gebreselassie and Antoniewicz (2015) proposed a framework determining the metabolic flux of multiple bacteria coexisting in a mixed system. ^13^CO_2_ and ^15^NH_4_ labeling strategies can be used to unravel the short-term flux of plant-assimilated C and fungal-obtained N through an *in situ* ectomycorrhiza system (Gorka et al., 2019).

Parsimonious MFA utilized a dual optimization minimizing both the SSE and the sum of the flux values. This minimization can be weighted by gene expression to integrate gene expression data with ^13^C data (Foguet et al., 2019). In COMPLETE-MFA, multiple substrates are used in parallel to generate complementary labeling information to improve the accuracy of the flux estimation. Generally, the accuracy can be significantly improved by utilizing 2–3 parallel data (Leighty and Antoniewicz, 2013). A truncated multi-model MCMC method was adopted to infer the *in vivo* probability of bidirectional reactions and to determine whether they are unidirectional or bidirectional (Theorell and Nöh, 2020). This can expand the range of ^13^C MFA from parameter inference to structure inference.

The reaction thermodynamic information from metabonomic and physiological data can restrict the solution space of flux and avoid *a priori* hypotheses about the flux direction (Saldida et al., 2020). With this tool, higher-precision inference of the network structure and flux values can be achieved, and some new flux patterns can be identified. Conversely, the Gibbs free energy of the reaction can be deduced from the metabolic flux values (Park et al., 2016).

## Application in neural cell research

Neurons and glial cells are the major cells of the nervous system. Neurons are a kind of specially differentiated cells with the ability to sense stimulation and conduct excitation, and is important to the functioning of the nervous system. Glial cells are not able to conduct impulses but nourish, insulate and protect neurons. Neurons provide electrical signaling to glial cells to fuel oxidative metabolism in the brain, while glial cells provide metabolic substrates to neurons. Metabolic activity in the brain requires a lot of energy. Cognitive phenotypes are related to neural metabolism, including neuronal mitochondrial mutation and neuron-glia metabolic crosstalk (Watts et al., 2018). Metabolic flux through both cell types is a factor modulating higher order phenotypes. Hence, it is rather interesting to discover how metabolic flux and neural cell function are related.

As outlined in Table 3, different types of ^13^C metabolic fluxomics have been successfully applied to study the metabolic reprogramming and its regulatory mechanism of neural cell (Lanz et al., 2013). INST-MFA was utilized to assess the metabolism shift upon differentiation of Neural Stem Cells (NSCs) into astrocytes, discovering an extensive decrease of central carbon metabolism and conversion of flux through TCA cycle to lac pathway during astrocytic differentiation (Sá et al., 2017). Isotope-tracing has been used to quantify the fraction of labeled monosaccharides in the glycans and glycosphingolipids of both pluripotent and neural NTERA-2 cells. It revealed that exogenous monosaccharide utilization would vary noticeably according to the cell differentiation state and different glycan structures (Wong et al., 2020). FR analysis and steady-state flux analysis was combined to find that more of glucose flux was channeled by glycolysis than that by pentose cycle of adherent cerebellar granule neurons. Meanwhile, it determined that 16% of glucose used by mitochondria comparing to 46% by lactate dehydrogenase (Gebril et al., 2016). By revising bi-directional reaction of the non-oxidative PPP pathway and TCA cycle, this method became more broadly applicable to different cell types (Jekabsons et al., 2017). Patel et al. (2021) investigated TCA cycle and neurotransmitter cycle fluxes ratio by FR method from a steady-state [2-^13^C] acetate experiment and the ^13^C turnover rates of neurotransmitter by a KFP-like fitting on labeling kinetics of amino acids in ^13^C glucose infusion experiment. The mitochondrial TCA flux of glutamatergic neurons and glutamate-glutamine cycle flux was declined in the cerebral cortex of aged mice. Isotope-tracing allows investigation of astrocyte-specific metabolic networks affected by Apolipoprotein (APOE) and observes an increase in flux through the pentose phosphate pathway, with subsequent increases in gluconeogenesis and lipid biosynthesis pathway in Apolipoprotein E4 astrocytes (Williams et al., 2020).

**TABLE 3:** Outline of neural cell studies using fluxomics method.

Tracer	Measurement target	Fluxomics method	References
[1-^13^C]glucose	Organic Acids, sugar phosphate and amino acids, such as malate, glutamine et al.	INST-MFA	Sá et al., 2017
[^13^C-U] monosaccharide such as glucose, galactose	N-glycan, O-glycan, and glycosphingolipids	Isotope-tracing	Wong et al., 2020
[1,2-^13^C2]glucose	Lactate	FR and SS-MFA	Gebril et al., 2016; Jekabsons et al., 2017
[1,2-^13^C2]glucose and Sodium [2-^13^C] acetate	Amino acids such as aspartate, GABA, glutamine	FR and KFP	Patel et al., 2021
[U-^13^C] glucose	Substrates of central carbon metabolic network, purine phosphates, uridine phosphates, glutathione, and et al.	Isotope-tracing	Williams et al., 2020

## Conclusion

Stable isotope metabolic flux analysis has been successfully used in applications from homogeneous cell systems to heterogeneous cell systems, even at the level of animal and plant organs. It has now become the gold standard for measuring metabolic flux values. Several technologies that it includes are also developing rapidly, so the metabolic fluxomics method is evolving into a family with different members that have higher accuracy, wider coverage, more application scenarios, and shorter time consumption.

At present, the development of stable isotope metabolic flux analysis should focus on improving the temporal and spatial resolution. This requires the continuous introduction of updated detection technology.

## Author contributions

BT, MC, and LL drafted the manuscript. TS and ZZ conceived of the study. TS, ZD, and BR helped to draft the manuscript. All authors read and approved the final manuscript.
